# Importance of endocrine treatment adherence and persistence in breast cancer survivorship: a systematic review

**DOI:** 10.1186/s12885-023-11122-8

**Published:** 2023-07-04

**Authors:** Finn Magnus Eliassen, Vibeke Blåfjelldal, Thomas Helland, Cathrine Fonnesbech Hjorth, Kari Hølland, Lise Lode, Bjørn-Erik Bertelsen, Emiel A. M. Janssen, Gunnar Mellgren, Jan Terje Kvaløy, Håvard Søiland, Tone Hoel Lende

**Affiliations:** 1 Department of Surgery, Stavanger University Hospital, PO Box 8100, 4068 Stavanger, Norway; 2 Hormone Laboratory, Department of Medical Biochemistry and Pharmacology, Haukeland University Hospital, Bergen, Norway; 3 Department of Clinical Science, University of Bergen, Bergen, Norway; 4 Department of Clinical Epidemiology, Department of Clinical Medicine, Aarhus University Hospital, Aarhus University, Aarhus, Denmark; 5 Division of Research, University of Stavanger, Stavanger, Norway; 6 Department of Gastrointestinal Surgery, Hvidovre Hospital, Copenhagen, Denmark; 7 Department of Pathology, Stavanger University Hospital, PO Box 8100, 4068 Stavanger, Norway; 8 Department of Chemistry, Biosciences and Environmental Engineering, University of Stavanger, Stavanger, Norway; 9 Department of Research, Stavanger University Hospital, PO Box 8100, 4068 Stavanger, Norway; 10 Department of Mathematics and Physics, University of Stavanger, Stavanger, Norway

**Keywords:** Breast Cancer, Endocrine Therapy, Adherence, Persistence, Survival

## Abstract

**Purpose:**

Adjuvant endocrine treatment is essential for treating luminal subtypes of breast cancer, which constitute 75% of all breast malignancies. However, the detrimental side effects of treatment make it difficult for many patients to complete the guideline-required treatment. Such non-adherence may jeopardize the lifesaving ability of anti-estrogen therapy. In this systematic review, we aimed to assess the consequences of non-adherence and non-persistence from available studies meeting strict statistical and clinical criteria.

**Methods:**

A systematic literature search was performed using several databases, yielding identification of 2,026 studies. After strict selection, 14 studies were eligible for systematic review. The review included studies that examined endocrine treatment non-adherence (patients not taking treatment as prescribed) or non-persistence (patients stopping treatment prematurely), in terms of the effects on event-free survival or overall survival among women with non-metastatic breast cancer.

**Results:**

We identified 10 studies measuring the effects of endocrine treatment non-adherence and non-persistence on event-free survival. Of these studies, seven showed significantly poorer survival for the non-adherent or non-persistent patient groups, with hazard ratios (HRs) ranging from 1.39 (95% CI, 1.07 to 1.53) to 2.44 (95% CI, 1.89 to 3.14). We identified nine studies measuring the effects of endocrine treatment non-adherence and non-persistence on overall survival. Of these studies, seven demonstrated significantly reduced overall survival in the groups with non-adherence and non-persistence, with HRs ranging from 1.26 (95% CI, 1.11 to 1.43) to 2.18 (95% CI, 1.99 to 2.39).

**Conclusion:**

The present systematic review demonstrates that non-adherence and non-persistence to endocrine treatment negatively affect event-free and overall survival. Improved follow-up, with focus on adherence and persistence, is vital for improving health outcomes among patients with non-metastatic breast cancer.

## Introduction

Breast cancer is the most common malignancy among women worldwide, with over 750,000 people diagnosed with early-stage breast cancer every year in the US and Europe, and 2.3 million worldwide [1–4]. It is a leading cause of death among women [5]. Moreover, it is estimated that there are 4.1 million breast cancer survivors in the US as of January 1^st^ 2022 [6]. Importantly, up to 75% of all breast malignancies are luminal subtypes, ie, tumors positive for estrogen receptor (ER) and/or progesterone receptor (PR) expression [7, 8]. For this type of breast cancer, most guidelines recommend adjuvant endocrine therapy (AET) after surgery and other adjuvant treatments. In premenopausal patients, this will involve 10 years of administration of the selective estrogen receptor modulator (SERM) tamoxifen. In postmenopausal women, aromatase inhibitors (AIs) are usually administered for 5 years to postmenopausal women [9]. In some regimens, the patient switches from an AI to a SERM [10].

The effectiveness of AET for breast cancer treatment is undisputed [11, 12]. The introduction of AET has reduced the rate of breast cancer relapse, and improved both breast cancer-specific survival and overall survival. Notably, the use of AIs has reduced breast cancer mortality during the first 15 years after diagnosis by approximately one-third [12–14].

Two important factors that jeopardize AET treatment success are non-adherence and non-persistence, which are two separate constructs describing different challenges [15]. In breast cancer, treatment non-adherence occurs when a patient fails to take the treatment as prescribed throughout the treatment period (ie, frequently missing doses), whereas non-persistence to AET occurs when a patient stops treatment continuously for a prolonged period of time.

For individual patients, adherence is usually reported as the percentage of days the prescribed dose of treatment is available to the patient over a specified period, ie, the medical possession ratio (MPR). This is commonly determined by analyzing prescription records. The threshold for categorizing a patient as adherent is generally set at an MPR of ≥ 80% [16]. Importantly, a large number of patients with breast cancer do not meet the conditions for adherence. On average, only 74% of patients with breast cancer adhere to AET [17, 18] during 5–10 years of breast cancer treatment, and adherence rates decrease over time [18].

In the breast cancer setting, non-persistence of endocrine therapy is described as a break in the continuous treatment with AET. The duration of a break required to classify a patient as non-persistent varies from 90–180 days [19, 20]. Persistence to AET varies from 31–73% after 5 years of treatment [21].

In this systematic review, we aimed to answer the research question how do non-adherence and non-persistence influence survival in women with breast cancer undergoing AET. This study may provide guidance for maximizing the beneficial effects of endocrine treatment in terms of the survival of breast cancer patients.

## Methods

This systematic review was conducted in accordance with the Preferred Reporting Items for Systematic Reviews and Meta-Analysis (PRISMA) statement and checklist, to ensure a complete and transparent report of our results.

### Eligibility criteria

The inclusion criteria were prospective and retrospective studies that investigated how patient adherence and/or persistence to AET were correlated with breast cancer recurrence and survival among patients with early-stage luminal breast cancer. We excluded studies on neoadjuvant treatment, prophylactic treatment, or subgroups of patients with ductal carcinoma in situ (DCIS), male only breast cancer, and metastatic breast cancer.

Previous publications have applied heterogeneous definitions of AET adherence and persistence [22]. From the included studies, we divided the results into two subgroups: non-adherence and non-persistence. Studies were included if they investigated the effects of AET non-adherence and/or non-persistence in women with early breast cancer. Moreover, the outcomes had to include breast cancer recurrence, disease-free survival, breast cancer-specific survival, or overall survival, with the former three end-points grouped as ‘event-free survival’.

A commonly used definition of adequate adherence is MPR ≥ 80%, which was used in most of the included studies. Although the optimal MPR cut-off point is not known, it is evident that relapses and mortality increase when MPR is below 80% [16, 23].

Persistence to medication use is calculated from the start of treatment until the patient completes the treatment or prematurely ends the treatment. Prescription databases are often used to monitor persistence, and a commonly used definition of non-persistence is the registration of a gap period without new prescriptions for several months (90–180 days) before the scheduled end of treatment [19, 24].

### Information sources and search strategy

Our systematic literature search was conducted on November 12^th^ 2020. Studies were retrieved from the following electronic databases: Medline (Ovid), Embase (Ovid), the Cochrane Library (CDSR and CENTRAL), CINAHL (EBSCOhost), and Web of Science. The search strategy was initially developed for Medline (Ovid) and appropriately adapted for other databases. The keywords of the Boolean search were "*breast cancer*" *AND* "*endocrine treatment*" *AND* "*adherence* OR *persistence*" *AND* "*recurrence OR survival*". Synonyms were used for all keywords (eg, compliance, refusal, etc.), and controlled vocabularies in the databases were used to extract all relevant search terms. Attachment 1 (Medline search strategy) presents a fully reproducible search strategy for Medline (Ovid). The search results were limited to English language articles published between 2010 and the search date. Conference abstracts and unpublished clinical trials were excluded.

**Fig. 1:**
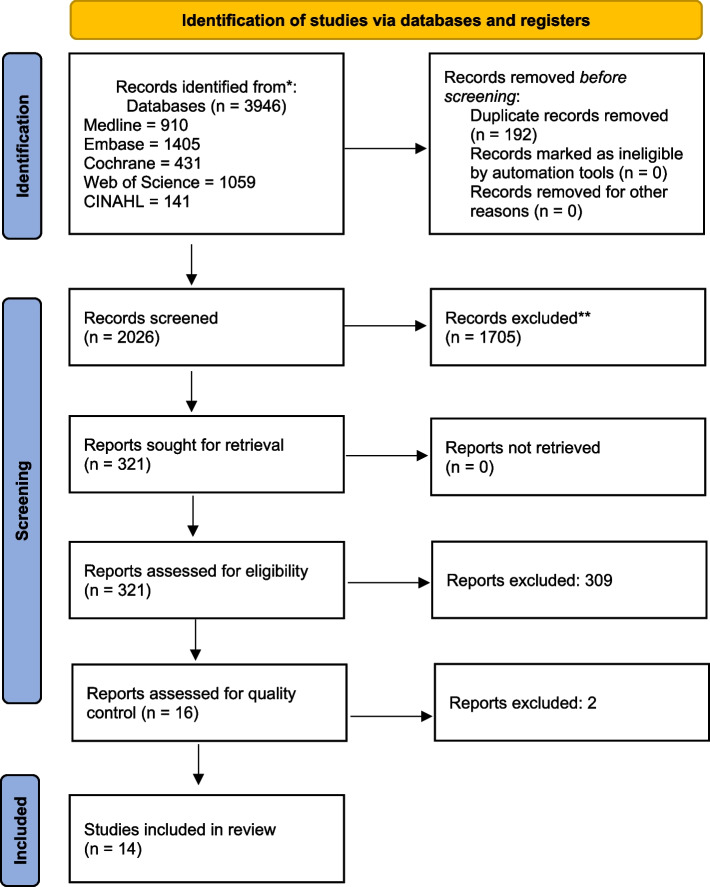
PRISMA flow diagram of the selection process to identify reliable studies.26. For more information, visit www.prisma-statement.org. From: Page MJ, McKenzie JE, Bossuyt PM, Boutron I, Hoffmann TC, Mulrow CD, et al. The PRISMA 2020 statement: an updated guideline for reporting systematic reviews. BMJ 2021;372:n71. https://doi.org/10.1136/bmj.n71

The search results were imported into EndNote X9, and duplications were removed. The final set of citations was imported into COVIDENCE [25] for further deduplication and eligibility screening of the titles and abstracts. Thus, the literature search, study design, and data analysis process followed the PRISMA guidelines [26]. All search results were uploaded, and titles and abstracts were independently screened by three authors (FE, HS, and TL), based on the predefined inclusion and exclusion criteria. These authors then conducted a full-text screening. Any conflicts were resolved by majority vote. Figure 1 shows a flow diagram of the selection process. Table 1 presents an overview of the study characteristics of the included studies. Table 2 includes an overview of the patient characteristics of the included studies.

**Table 1:**
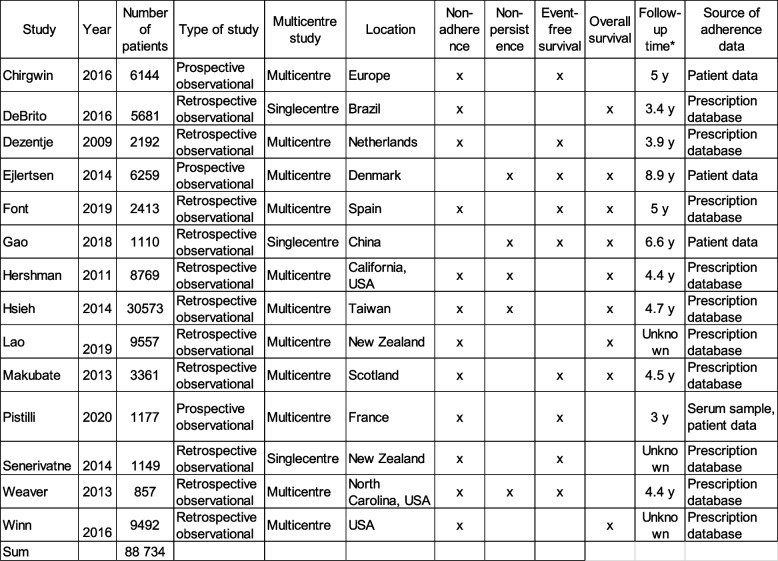
Overview of the studies included in the systematic review

^*^Follow-up time is defined as the time from the first treatment dose and last follow-up or patient censoring

**Table 2:**
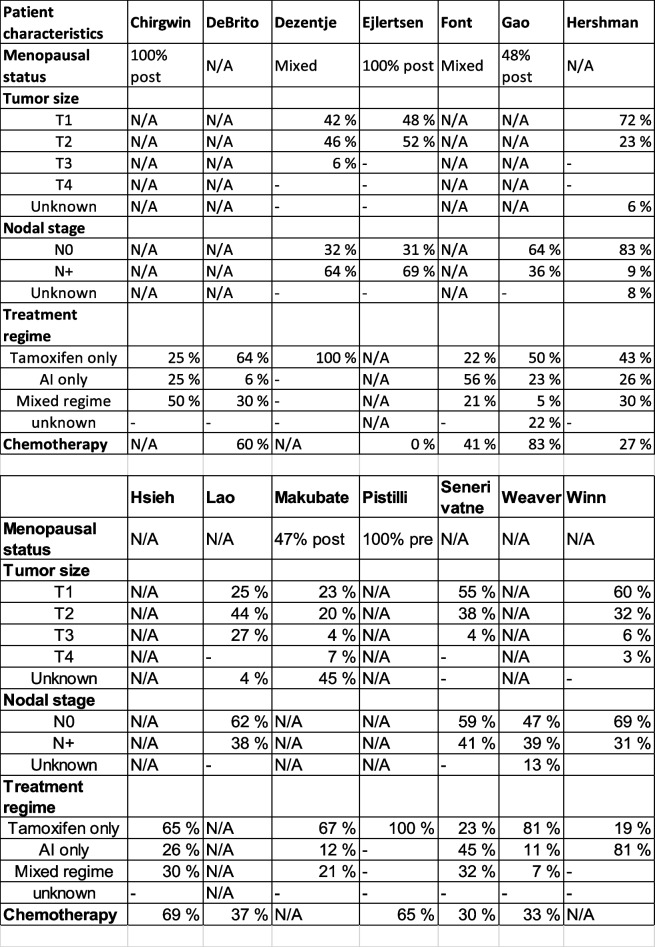
Patient characteristics of the included studies

*N/A* Not available

The included studies were evaluated for risk of bias using the Cochrane Risk of Bias Comparison in Covidence. The individual studies were graded as having high, low, or unclear risk of bias in the following categories: sequence generation, allocation concealment, blinding of participants and personnel for all outcomes, blinding of outcome assessors for all outcomes, incomplete outcome data for all outcomes, selective outcome reporting, and other sources of bias. Table 3 presents a full description of the bias analysis.

**Table 3:**
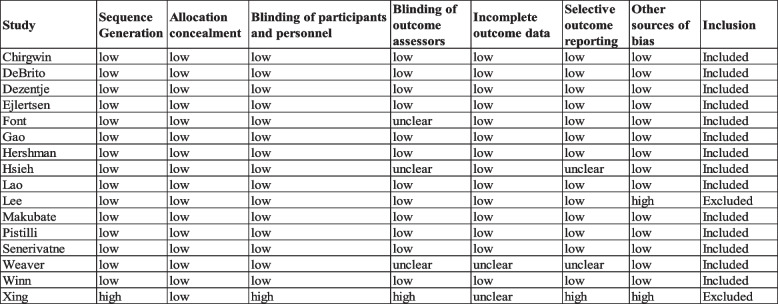
Quality assessment of the studies assessed for the systematic review

### Statistical analysis

Hazard ratios (HRs) were used to describe the relative risk of non-adherence and non-persistence to AET. Forest plots were generated to present HRs with calculated 95% confidence intervals (CIs) and the number of patients. Variables were coded such that the HR represented the relative risk of non-adherent/non-persistent patients versus adherent/persistent patients. We separately analyzed event-free survival and overall survival. The analyses were performed using R version 4.2.1 [27]. The studies exhibited substantial heterogeneity in study design, analysis approaches, definitions of adherence and persistence, whether adherence was treated as a time-dependent covariate, definitions of outcome variables, and adjustment for specific factors. This implies that the HRs reported in the different studies are not directly comparable, as they measure different quantities and have different interpretations. Consequently, it was not appropriate to perform a meta-analysis, even though the individual HR values were independently valid.

## Results

After removing duplicates, 2,026 papers were retrieved from various databases. Among these papers, 321 articles were reviewed in full text, and 16 studies met our inclusion and exclusion criteria for quality review [4, 19, 20, 24, 28–40]. Of these 16 studies, one was excluded because its study design implied immortal time bias [40], and another was excluded because it used a time variable that changed the patient selection to exclude patients with breast cancer events during the first 4.5 years [39]. Ultimately, 14 studies examining different patient cohorts met the eligibility and quality criteria according to the PRISMA guidelines and Cochrane Risk of Bias comparison, to assess how patients’ non-adherence and/or non-persistence to endocrine treatment was associated with prognosis. Among the 14 included studies only ten explicitly state that patients with a previous breast cancer have been excluded [19, 24, 28, 30–34, 37, 38]. This is not explicitly stated in the remaining four studies [17, 20, 29, 36].

The measured outcome variables differed among the studies, and included local relapses, distant metastases, breast cancer deaths, and overall survival (Table 1). Eleven studies analyzed the effects of patient adherence, and in eight of these articles non-adherence was defined as an MPR < 80% [19, 24, 28, 29, 33, 35–38]. One study used non-dichotomized MPR during the first year of treatment as a time-invariant predictor [20]. Another study used serum measurements after 1 year of treatment as a proxy for adherence [34]. In another study, the authors defined adherence as taking at least 90% of the dispensed packs as prescribed [31].

Six studies analyzed patient persistence. Three studies defined non-persistence as discontinuation prior to 4.5–5 years of treatment [30, 32, 41]. Non-persistence was defined as a 180-day gap between prescriptions in two studies [19, 29], and as a 90-day gap between prescriptions in one study [20].

Among the 14 studies, four used patient-recorded data to analyze adherence and persistence, ten studies examined prescription records, and one used serum samples in addition to patient data. The sample size ranged from 857 to 30,573.

**Fig. 2:**
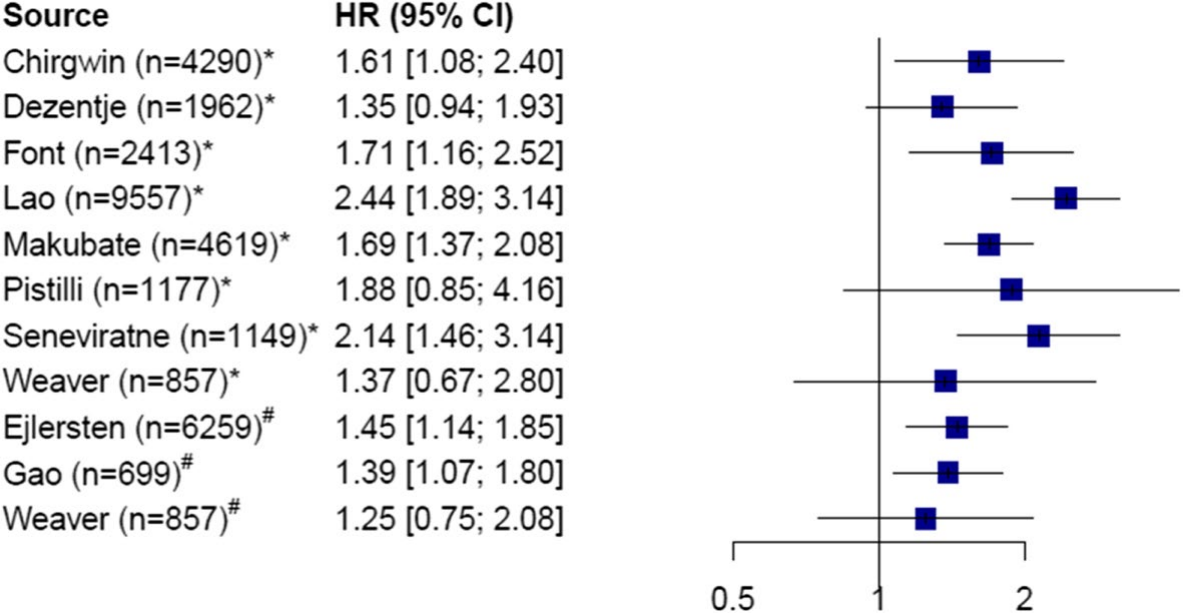
Event-free survival Forest plot for event-free survival, reporting the hazard ratio (HR) for non-adherent (*) and non-persistent (#) patients with breast cancer. n = number of patients in the study

Eight studies measured the effect of non-adherence, and three measured the effect of non-persistence on event-free survival. Among these studies, seven showed significantly poorer outcomes in the non-adherent and non-persistent groups. A full overview of the available HRs is shown in Table 4 and Fig. 2. The HRs ranged from 1.39 (95% CI, 1.07 to 1.53) to 2.44 (95% CI, 1.89 to 3.14). The remaining four studies showed the same tendency; however, the results were not significant because of large confidence intervals, partly due to the inclusion of fewer patients (Fig. 2).

**Table 4:**
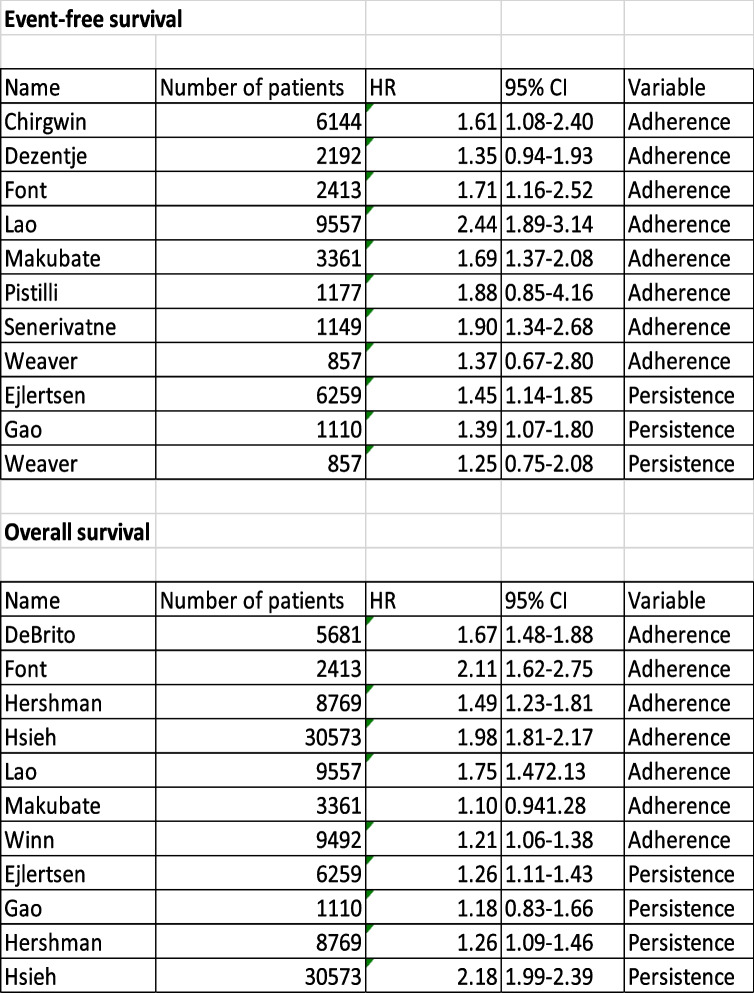
Hazard ratios (HRs) with 95% confidence intervals due to non-adherence and non-persistence

**Fig. 3:**
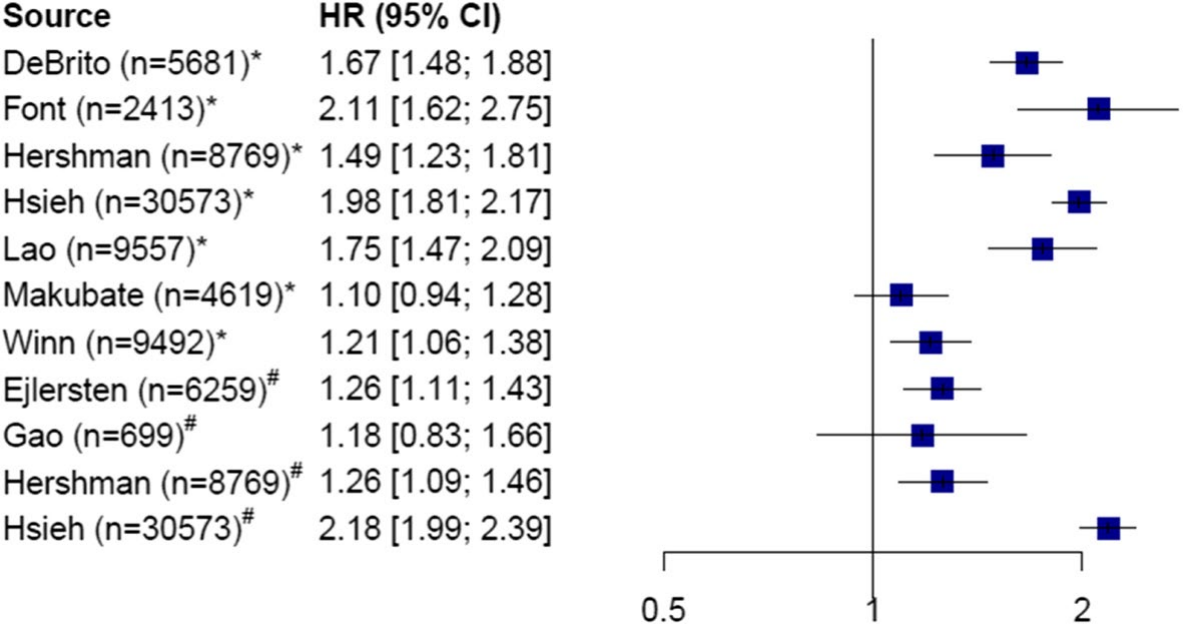
Overall survival. Forest plot for overall survival, reporting the hazard ratio (HR) for non-adherent (*) and non-persistent (#) patients with breast cancer. n = number of patients in the study

Nine studies investigated how non-adherence or non-persistence was associated with overall survival. Seven of these studies showed that poor adherence and/or persistence was associated with significantly reduced overall survival, with HRs ranging from 1.21 (95% CI, 1.06 to 1.36) to 2.18 (95% CI, 1.99 to 2.39). The remaining studies showed the same trend, but with non-significant results (Fig. 3).

Of the 14 studies, seven reported tumor size, eight reported nodal stage, twelve reported treatment regime and ten reported use of chemotherapy. The reported patient characteristics can be found in Table 2.

The largest analyzed study [19] was a retrospective cohort study from Taiwan, which included 30,573 patients, collected from the Taiwan National Health Insurance Research Database (NHIRD), which covers 99.6% of the population in Taiwan. Their results showed that non-adherence led to decreased event-free survival, with an HR of 1.98 (95% CI, 1.81 to 2.17), and non-persistence was associated with decreased event-free survival (HR: 2.18; 95% CI, 1.99 to 2.39). The rates of relapse and death were effectively doubled in the populations that did not use AET as prescribed.

The reviewed studies clearly show a marked increased risk of relapse or death due to non-adherence and non-persistence to AET, highlighting the importance of this treatment for women with non-metastatic luminal breast cancer.

## Discussion

The present study is the largest systematic review on the prognostic role of non-adherence and non-persistence to AET among patients with early-stage breast cancer, since 2010. We screened over 2,000 published studies and identified 14 studies that met our clinical standards for reliable analysis.

Although there was a substantial heterogeneity in the design of the included studies, the forest plots clearly depicted a trend of higher rates of adverse events among the non-adherent and non-persistent patients. The available data highlight the dangers of non-adherence and non-persistence, showing an up to two-fold higher risk of relapse or death for patients who do not use endocrine treatment as prescribed.

A systematic review published in 2021 reviewed 12 studies and included only studies published during the past five years and available in PubMed. It also found that non-adherence and non-persistence to endocrine treatment had a marked negative effect [42]. Of the 12 studies included in that review, 7 were also included in our present review, while the remaining five did not meet our quality criteria.

Of the included 14 studies, 6 were located in Europe, three were located in USA, two were located in New Zealand, two were located in East Asia and one was located in Brazil. This evident skew towards first world countries is an unfortunate, but unsurprising find. Adherence and persistence are issues with many socio-economic and cultural causes, and the fact that research is primarily done in first world countries limit the finding to these select groups. Similar research should be conducted on a global scale to allow for cultural and geographical differences as breast cancer is a global issue, and AET is used world-wide.

### Treatment of side-effects

There is a close relationship between adverse events, and patient adherence and persistence. This point has been raised in several large multi-centre studies such as BIG-1–98 [31] and MA.27 [43]. Medication side-effects are believed to play a major role, where symptoms that are caused by depletion of circulating estrogens such as hot flashes [44], muscle pains and vulvovaginal symptoms are commonplace [45]. The women may seek to minimalize these side-effects by either reducing adherence or persistence. Despite this fact, there is a history of underreporting side-effects by clinicians [46]. When asked, 81% of all breast cancer patients on AET report at least one major side-effect, and the majority report more than one.

The patients who report side-effects have a significantly increased risk of non-persistence to treatment [47]. Musculoskeletal symptoms are one of the most reported side-effects among patients undergoing AI treatment. It is suggested that these side-effects cause premature discontinuation, and a study of 3887 patients showed 15% non-persistence after 12 months of treatment, with 63% citing musculoskeletal symptoms as the cause of non-persistence. Despite several high quality studies showing a significant relationship between AET side-effects and non-adherence and non-persistence, the large variation in study characteristics and definitions of adherence makes it difficult to draw any definite conclusions as to the causes of non-adherence [48].

An important factor to take into consideration is that despite it being understandable that some breast cancer survivors may stop adhering to their treatment if the side effects are intolerable, there may be ways to reduce the side effects, for example vasomotor drug treatment, switching between different AETs, or non-pharmacological approaches [44, 49]. Although there are many treatments available to reduce side-effects, only 44% of patients with side-effects use medications to overcome them [47]. There is evidently a large variation in the intensity and type of side-effects in this patient group. Having treatment guidelines in place, with individualized treatment plans for managing these side effects, can be one factor in improving patient adherence.

There may be a direct link between patient side-effects and the effect of AET on survival outcomes, where patients with vasomotor side-effects when undergoing AET have been shown to have a greater decrease in breast cancer recurrence than patients with no side-effects [50]. Similarly in a study of the WHEL trial, where 864 patients were taking tamoxifen, the patients who reported hot flashes at baseline were less likely to develop recurrent breast cancer after 7 year follow up [51]. This paradoxical effect of higher survival rates in a patient group with lower expected adherence may indicate a greater response to AET in the group of patients that experience larger side effects of medication. Building on this fact, individual assessments of active tamoxifen metabolites could be used to predict side effects [52] and survival [45, 53]. This point further highlights the importance of continuing AET despite its troublesome side-effects.

The four-way link between serum levels of active tamoxifen metabolites, medication side-effects, non-adherence and persistence, and survival outcomes among these patients is therefore a better approach for increased knowledge of the factors that can improve patient outcome in breast cancer patients undergoing AET.

### Sequential treatments

Planned sequential treatment (two years AI followed by three years tamoxifen, or two years tamoxifen followed by three years of AI) have been studied in a large randomized trial against five years of AI and five years of tamoxifen. The rates of non-persistence have been shown to be poorest among the patients who have received sequential treatment [31]. This suggests that patients find it difficult to handle a new set of side-effects after a switch in treatment. In long-term follow up studies on this cohort of patients, the AI arm has shown a relative risk reduction of 9% after 12.6 years [54]. Despite this, for a subset of patients with a high burden of adverse effects, switching from AI to tamoxifen may be preferable to stopping all treatment [54]. Several other studies have found that switching regimens from tamoxifen to AI may increase compliance, and can be beneficial to survival [55]. Individual treatment plans, and an understanding of each patients challenges with adverse events and side-effects is therefore important when determining the treatment plan.

### Socioeconomic positions

Demographic variables and patients’ psychological and financial situations are reported to be important predictors of poor adherence [56, 57]. In a nested cohort study including 2,616 patients from Denmark, patients with low socioeconomic position (SEP) were found to have increased 5-year cumulative incidence rates of disease relapse (13%) and breast cancer-related death (11%). This study also found that single women had increased 5-year rates of recurrence (RR, 1.45; 95% CI, 1.11 to 1.89) and mortality (RR, 1.83; 95% CI, 1.32 to 2.52) [58]. The socioeconomic disparity was most evident within the group of patients with ER + tumors, suggesting that lower treatment adherence in these groups may partly explain the differences in survival and recurrence. Further studies of how socioeconomic factors are related to outcome could potentially enable the identification of subsets of patients who have an increased risk of non-adherence, and who thus require closer follow-up throughout their treatment.

### Global challenges

Medicine availability remains a global challenge. Breast cancer is a major health concern in poorer areas of the world and, for many people, the lack of availability and prohibitive cost of medication leads to poor adherence and non-persistence. Women must shoulder the double burden of disease and inadequate healthcare, which broadens the health gap and increases disparities. In the United States, it has been reported that racial and ethnic diversity impacts treatment and survival [59]. Since these disparities are multifactorial, the strategies aimed at reducing them must involve advocacy, research, education, and healthcare services. The success of these strategies depends on their support at the federal and state levels, and the involvement of local communities in developing programs and policies that are culturally and linguistically appropriate for their communities, in order to ensure both the utility and the duration of these efforts [59].

### Strengths and limitations of this study

The present study has several limitations. First, only a small number of studies were included in this analysis, despite over 2,000 studies being reviewed. This may be due to a lack of research focused on adherence to adjuvant endocrine treatment in the breast cancer research community and may be an indication of the complexity of endocrine treatment adherence. Additionally, studies were excluded from the analysis due to problems related to bias and study protocols that made the results unreliable. Despite the high quality of the 14 included studies, the heterogeneity was too great to allow us to perform a meta-analysis. This was mainly due to the use of different definitions for adherence and persistence, differing outcomes and analysis methods, and differences in follow-up time and the treatment received, which make it difficult to provide a summated HR with 95% CI.

Implementing common definitions of adherence and persistence would expand the possibilities of studying the effects of poor adherence to AET. There is presently no gold standard for assessing adherence to AET therapy. Using an MPR cut-off of 80% adherence seems to be the most widely used definition of adequate adherence to AET and can be feasibly measured using pill counting or prescription records. However, this is a measurement with many potential sources of error. On the other hand, more direct approaches, such as serum measurements of active metabolites, can provide a more objective status, but have yet to be accessible for large-scale studies [34, 60, 61].

Reporting of tumor size and nodal status was lacking in several of the studies. This gives limitations as to which patients are most affected by non-adherence and non-persistence. Standardized reporting of patient characteristics would allow for better comparisons of the patient groups included in different studies and can give evidence as to which patient groups suffer more from non-adherence and non-persistence.

### Further research and measures

Our present review indicated that non-adherence and non-persistence to AET has been a neglected research topic for decades. Importantly, improving adherence and persistence represents a low-hanging fruit for increasing survival in luminal breast cancer. There is a need for tailored, interdisciplinary, long-term follow-up strategies, including identification and treatment of side effects. Moreover, serum detection of tamoxifen and AI metabolites during the whole treatment period would enable identification of non-adherent patients, and allow doctors to adjust treatment to more functional levels to reduce side effects [45, 61]. Establishing AET units in oncological/surgical departments to promote cooperation between clinicians, family doctors, oncology nurses, psychiatrists, psychologists, and social workers may help to ensure that patients receive the care that they need to improve adherence [58]. However, randomized studies are needed to evaluate how such AET units impact adherence and survival. If effective, AET units could be a low-cost, high-reward means of improving the survival of women with breast cancer.

## Data Availability

All data generated or analyzed during this study are included in this published article.

## Abbreviations

HRs: Hazard ratios
ER: Estrogen receptor
PR: Progesterone receptor
AET: Adjuvant endocrine therapy
SERM: Selective estrogen receptor modulator
AIs: Aromatase inhibitors
PRISMA: Preferred Reporting Items for Systematic Reviews and Meta-Analysis
DCIS: Ductal carcinoma in situ
CIs: Confidence intervals
NHIRD: Taiwan National Health Insurance Research Database
SEP: Socioeconomic position
